# Effects of an Internet-Based and Teacher-Facilitated Sexuality Education Package: A Cluster-Randomized Trial

**DOI:** 10.3390/children8100885

**Published:** 2021-10-03

**Authors:** Zhao Jin, Fuyu Guo, Kai Wang, Hanxiyue Zhang, Wenzhen Cao, Jiayi Hee, Yuan Yuan, Minne Chen, Kun Tang

**Affiliations:** 1 Vanke School of Public Health, Tsinghua University, No. 30 Shuangqing Road, Beijing 100084, China; jinzhao111@pku.edu.cn (Z.J.); fyguo@pku.edu.cn (F.G.); kaiwang_application@outlook.com (K.W.); hxyz@mail.tsinghua.edu.cn (H.Z.); j.hee@uqconnect.edu.au (J.H.); 2 China-Japan Friendship Hospital, No. 2 Sakura Garden East Street, Chaoyang District, Beijing 100029, China; 3 Department of Epidemiology, T.H. Chan School of Public Health, Harvard University, Boston, MA 02115, USA; 4 Shantou University Medical College, No. 22 Xinling Road, Shantou 515041, China; caowz@pku.edu.cn; 5 School of Public Health, Shantou University, No. 243 Daxue Road, Shantou 515063, China; 6 School of Public Health, Peking University, No. 38 Xueyuan Road, Beijing 100191, China; yuanyuan@bjmu.edu.cn; 7 Department of Sociology, University of North Carolina at Chapel Hill, 103 S Bldg Cb 9100, Chapel Hill, NC 27599, USA; mchen16@live.unc.edu

**Keywords:** sexuality education, internet-based education, sexual knowledge, sexual attitudes

## Abstract

Background: This study aimed to evaluate the effects of an internet-based and teacher-facilitated sexuality education package on the sexual knowledge and attitudes of Chinese adolescents. Methods: Six middle schools where no sexuality education had been performed with a total of 501 adolescent students (245 males and 256 females) were included in the trial. In total, 14 classes were randomly assigned to the intervention (internet-based sexuality education package) or the control group (classes were conducted as per normal). Students’ sexual knowledge and attitudes were assessed at the baseline, at the end of the intervention, and 12 months after the intervention. Generalized linear models were employed to assess the effects of the intervention. Results: Positive effects of the intervention were observed on sexual knowledge (β = 4.65, 95% CI: 4.12—5.17) and attitudes (β = 1.25, 95% CI: 1.00—1.50) at the end of the intervention. After 12 months, the effects sustained but the magnitude declined for sexual knowledge (β = 2.39, 95% CI: 1.85—2.93) and attitudes (β = 0.49, 95% CI: 0.23—0.75). There were no significant differences between male and female students. Conclusions: Although further modifications are required, the sexuality education package can increase the accessibility of comprehensive sexuality education to adolescents in rural areas in China.

## 1. Introduction

China has seen greater acceptance and tolerance toward sexuality and sexual behaviors [1]. An increase in the proportion of sexual practice among Chinese college students was found over the past 30 years [2,3]. However, due to the lack of sexual knowledge, Chinese adolescents, especially those from undeveloped rural areas, were found to be susceptible to adverse sexual health outcomes [4]. Unintended pregnancies, abortions, and sexually transmitted infections (STIs) have been of increasing health and social concern [5]. According to a nationwide survey, 6.3% of unmarried young women aged 15–24 years reported unwanted pregnancies, and 82.5% of them ended in abortions [6]. Moreover, STIs, such as chlamydia and gonorrhea, have surged among Chinese youth and young adults dramatically during the last several decades, with a 16% annual increase in prevalence [7,8].

Comprehensive sexuality education plays an important role in preventing adverse sexual and reproductive health outcomes [9,10,11]. Comprehensive sexuality education is beyond teaching adolescents about the anatomy and physiology of sexual reproduction. Healthy sexual development, gender identity, and interpersonal relationships should be covered, as suggested by the United Nations Children’s Fund (UNICEF) [12]. Effective sexuality education during puberty can enable adolescents to make sensible decisions and reduce risky sexual behaviors, beneficial in promoting better sexual and reproductive health [13].

Although the Chinese government formally introduced sexuality education into the schools’ curricula in 1988 and has regarded it as an age-appropriate prevention strategy against acquired immunodeficiency syndrome (AIDS) and other STIs [12], sexuality education in schools in China is still insufficient [14]. It was reported that only 55.6% of college students have ever received school-based sexuality education [15]. Several reasons might account for this lack of sexuality education. First, in China, educators and parents traditionally shy away from discussing sex-related topics, and some may even regard sex-related topics as taboo subjects [16,17]. Second, considering that there have been no formal curriculums on sexuality education, and no professional educators trained in addressing this subject, schools have difficulty in designing sexuality education packages. Third, students may tend to perceive sexuality education as a low-priority and non-examinable subject and would rather invest more time in learning other compulsory courses [18]. The situation is further exacerbated in undeveloped regions where students have fewer educational resources [19]. Last, although a study in Europe has reported reduced violence and greater respect for sexual diversity through educational intervention [20], such issues have been rarely introduced in the current sexuality education curriculum in China.

To counteract the challenges of traditional sexuality education, an internet-based and teacher-facilitated educating platform “You and Me” was developed by Marie Stopes China to provide free and standardized comprehensive sexuality education to adolescents [21]. All contents in the platform were developed by experts in sexual and reproductive health in China. Internet-based sexual health promotion has been demonstrated to be effective in influencing psychosocial outcomes and increasing sexual knowledge [22]. Furthermore, compared with other internet-based programs, the sexuality education packages developed by Marie Stopes China do not exclude school teachers. Teachers are supposed to play an active role in ensuring the complete delivery of the course sessions and facilitating the teaching process. To make it easier for teachers to deliver the sexuality education package, each session in the package has been designed to be 40 to 45 min long, equaling to the length of a usual class. The integration of internet-based sexuality education into traditional school education is expected to largely improve quality and accessibility, and, thus, also improve the outcomes of sexuality education in economically disadvantaged regions of China.

On the platform, a special package designed for middle school students is provided. However, the effectiveness of the package has never been assessed. To evaluate the effectiveness of this internet-based and teacher-facilitated sexuality education package, we conducted a cluster-randomized trial in Longnan, Gansu Province, the poorest administrative division in China [23].

**Hypothesis** **1**. *The objective of this study was to assess the change in students’ sexual knowledge and attitudes after completing the courses in the package. In this study, it was hypothesized that students’ scores of sexual knowledge and attitudes would increase in the intervention group but remain roughly the same in the control group. If scores of the control group were observed to increase, the effectiveness of the intervention can be demonstrated as long as the magnitude of increment was larger in the intervention group than in the control group*.

## 2. Materials and Methods

### 2.1. Design, Setting, and Participants

The study design is a cluster-randomized trial with equal randomization at the cluster level. In China, middle schools include junior middle schools and senior middle schools, covering adolescents of 12 to 18 years. Six junior middle schools located in Longnan, Gansu Province, without formal sexuality education were selected as clusters in this trial. The inclusion criteria for the trial were seventh-grade students who had consent from their parents or guardians. In the intervention group, internet-based sessions from the sexuality education package were delivered during classroom hours by teachers once a week over 2 months, and in the control group, classes were conducted as per normal. No changes to the trial protocol were made during the trial. The study was approved by the Tsinghua University Institutional Review Board (IRB) (Project No: 20190009).

### 2.2. Sexuality Education Package Content and Delivery

The sexuality education package for middle school students on the “You and me” platform consists of eight 45 min sessions, titled “Knowing Sexuality Education”, “Knowing Gender”, “The Reproductive System”, “Puberty”, “Pregnancy and Contraception”, “Sexual Infection and Behavior”, “Knowing Sexual Violence”, and “Romantic Relationship and Marriage”. Each session contains well-developed handbooks, scripts, and course slides that teachers could utilize to facilitate the courses. Online videos on cartoons that teach students standardized knowledge on sexual and reproductive health were also included in each session of the package. All contents were developed by experts in sexual and reproductive health in China. Teachers were trained by research staff members before they delivered and facilitated this internet-based course during classroom hours. To familiarize teachers with the contents of the sexuality education package and to improve teaching skills, video recordings of trained educators conducting each session were provided as references.

### 2.3. Assessments

When designing the assessment on the participants’ sexual knowledge and attitudes, we adopted the Global Early Adolescent Survey (GEAS) and followed the guideline in Questionnaire Design in Reproductive Health Epidemiology provided by the US Centers for Disease Control and Prevention (CDC) [24]. The GEAS is a commonly used measurement instrument when studying early adolescents, and the validity of the gender norm section in the GEAS has been proven [25]. Necessary customizations were made to make the items easier to be implemented and to be understood by participants in the context of Chinese society.

Sexual knowledge was measured by 14 true-or-false questions comprising topics on puberty, gender-based violence, pregnancy and contraception, abortion, and sexually transmitted infections (Supplementary Material Table S1). One point was assigned if a question was answered correctly. The scores range from 0 to 14, with high scores indicating thorough sexual knowledge.

Sexual attitudes were measured by 14 items using Likert-type answer scales consisting of topics on discussing sex, gender equity, gender-based violence, homosexuality, and seeking help (Supplementary Material Table S2). For each item, the response ranged from “strongly agree” (=0) to “strongly disagree” (=4). We identified conservative and liberal attitudes for each item according to the United Nations Population Fund (UNFPA) technical guidance [26]. For the 10 regular items (item 1, items 3–10, item 13, and item 14), “strongly disagree” (=4) were identified as liberal. For the other 4 items (item 2, item 11, item 12, and item 14), “strongly agree” (=0) were identified as liberal. These four items were subtracted from 4 to reverse score them. The total attitudes score measured the degree of liberalism, with high scores representing liberalism. The original attitudes score ranged from 0 to 56. To make the scale range consistent with the knowledge scores, the total attitudes score of each participant was divided by 4. Hence, the scaled attitudes score also ranged from 0 to 14. The sexual practice was not studied in this study because of the low prevalence among the participants. Even in the last follow-up survey, only 12 participants (2.80%) reported they have ever had sexual practice. Thus, the effects of the intervention on sexual practice and its related outcomes (e.g., unintended pregnancy and STIs) were not studied in this trial.

Surveys were conducted at baseline, end of the intervention, and 12 months after the intervention by investigators from Tsinghua University during school hours to collect information on students, as well as to assess sexual knowledge and attitudes. Students were instructed by investigators to submit their answers using an online survey platform (SojumpTech Co. Ltd., Shandong, China). Investigators were responsible for checking data completeness.

### 2.4. Sociodemographic Characteristics

Baseline sociodemographic information on each student—namely, sex (Male or Female), age, hukou (Rural, Urban, or Unknown), ethnicity (Han or Others), whether they have had sexual practice before the baseline survey (Yes or No), and whether their parents were divorced (Yes or No), were also collected as covariates in this trial. The existing literature confirms that these covariates are associated with sexual knowledge and attitudes [1,27].

### 2.5. Randomization

The study was a clustered randomized trial, and randomization was conducted at the school and classroom levels. The study employed two stages of randomization. At the first stage, the six eligible schools were randomly assigned to either intervention or control arms using a program-generated random number by an investigator from Tsinghua University. At the second stage, two or three classrooms per school were randomly selected for the trial. Seven classrooms from the three schools assigned to the intervention arm were selected, and seven classrooms from the three schools assigned to the control arm were selected. The nature and the purpose of the trial were explained by investigators. Students with parental consent were included in the study. A teacher in the classroom was responsible for collecting parental consent. Parental consent forms were kept by the research staff.

### 2.6. Blinding

Due to the nature of the intervention, neither participants nor statisticians were blinded to the assignments. Investigators who conducted the baseline survey and delivered the follow-up tests were blinded.

### 2.7. Statistical Analysis

Participants’ demographic characteristics at baseline between the two arms were compared. Descriptive analyses were also conducted to assess attrition rates between the intervention and control arms, and to examine whether participants lost to follow-up had different characteristics, compared with those remaining in the trial. Categorical variables were compared using the chi-square test, and continuous variables were compared using a *t*-test.

A generalized linear model (GLM) for repeated measurements analysis was performed to examine the intervention’s effects on students’ sexual knowledge and attitudes. The model includes a term indicating the intervention group, two terms indicating the survey round, and their interaction terms. The coefficients of the interaction terms represent the patterns of change over time between the intervention and control arms, which are the intervention effects in the trial. Demographic characteristics were adjusted as fixed effect terms. For the dependency of repeated observations within an individual and the interdependency of participants within a school, two random intercepts representing individual and school were added to the model. Although some studies suggested excluding the intervention term from the model [28], we kept it as a fixed effect term in the main analysis and then excluded it during the sensitivity analyses. To explore the potential interaction effect of intervention and sex, subgroup analysis by sex was conducted, adjusted for the same variables in the main analysis.

To avoid potential issues related to multiple testing, the maximum probability of type I error was calculated and reported under the condition that two null hypotheses were true (i.e., the intervention has no effects on adolescents’ sexual knowledge, and it has no effects on adolescents’ sexual attitudes). To assess the robustness of the results, sensitivity analyses were performed. First, GLM was performed without the intervention term. Second, the analysis was restricted only to students who completed all three surveys to test the effect of students lost to follow-up on the association. All data processing and statistical analyses were performed using R 4.0.0 (R Core Team, 2020).

## 3. Results

### 3.1. Participants and Recruitment

**Figure 1:**
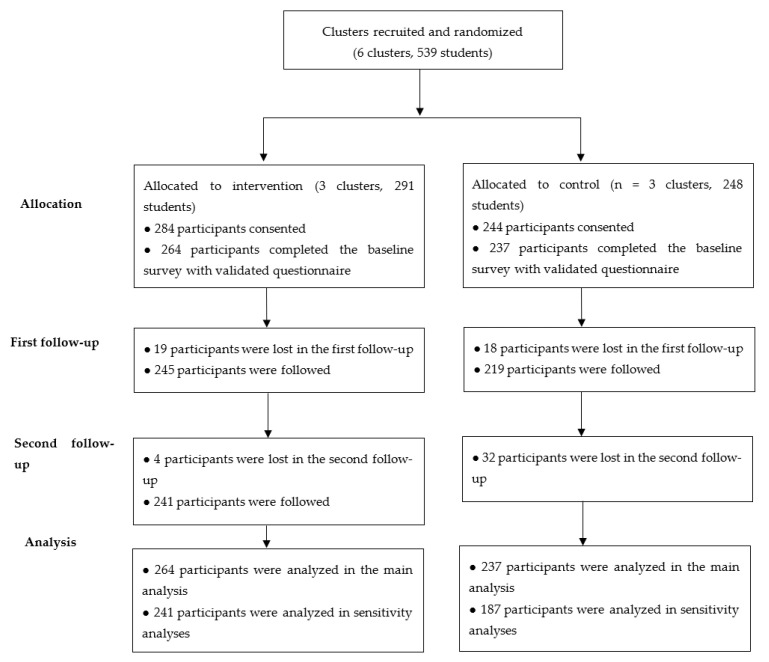
Trial flow diagram.

Figure 1 presents the flow diagram of the inclusion and exclusion of students. A total of 539 students from 6 schools were invited to participate in the trial between 21 October 2018 and 25 October 2018. Parents or guardians of 284 students (response rate = 97.59%) in the intervention group and 248 students (response rate = 98.39%) in the control group gave consent for participation. Students without their parents’ consent were excluded from the intervention and the sexuality education courses. The baseline survey was conducted as soon as the recruitment in the cluster was complete. The intervention ended when the class completed all the eight sessions in the package. The first follow-up survey was performed between 23 December and 27 December 2018. The second follow-up survey was conducted between 23 December and 24 December 2019.

### 3.2. Baseline Survey

The characteristics of participants at baseline are presented in Table 1. Except for participants’ age (*p* = 0.012) and hukou (*p* < 0.001), there were no significant differences between the two arms. On average, participants in the intervention group were 0.2 years older than participants in the control group. More participants in the intervention group reported their hukou as unknown, compared with participants in the control group (16.29% vs. 7.59%), whereas more participants in the control group reported their hukou as urban, compared with participants in the control group (27.85% vs. 15.91%). The proportion of participants with rural hukous were similar in the two intervention arms (67.80% vs. 64.56%). The majority of participants were Han Chinese (*n* = 491, 98.73%), and reported never having had sexual practice before the baseline survey (*n* = 493, 98.48%). About one-tenth of participants (*n* = 55, 10.98%) reported their parents divorced. At baseline, the intervention group demonstrated lower average knowledge scores (3.81 vs. 4.48, *p* = 0.004) and lower average attitude scores (7.73 vs. 7.98, *p* = 0.016), compared with the control group.

### 3.3. Analyzed Number and Attrition

At baseline, 264 participants in the intervention group and 237 participants in the control group completed the survey and were included in the main analysis. However, 12 months after the intervention, only 241 participants in the intervention group (retention rate = 91.29%) and 187 participants in the control group (retention rate = 78.90%) remained, with a significant difference between the two arms (*p* < 0.001).

Table 2 presents the characteristics of participants according to those who remained in the trial, and those individuals who were lost to follow-up. Students lost to follow-up were more likely to be male (69.86% vs. 45.33%, *p* < 0.001), older (13.1 vs. 12.8, *p* < 0.001), reported their parents divorced (21.92% vs. 9.11%, *p* = 0.002), and demonstrated lower attitude scores at baseline (7.52 vs. 7.91, *p* = 0.003). No significant differences between students who remained and students who were lost to follow-up with regard to their sexual practice status and baseline sexual knowledge scores.

### 3.4. Knowledge and Attitudes Outcome

**Figure 2:**
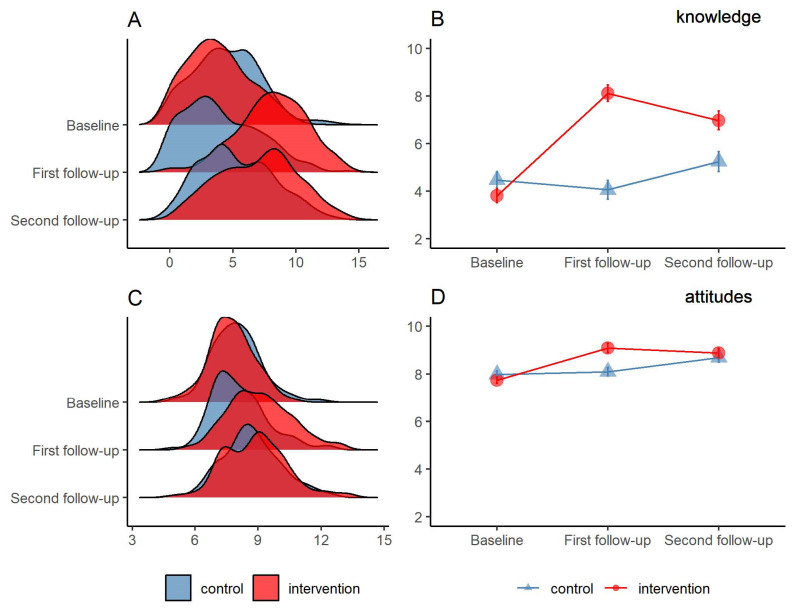
Student’s sexual knowledge and attitudes score in three rounds of surveys by intervention arm (N = 501). (**A**)The ridge plot comparing sexual knowledge score distribution among baseline and the following two follow-up tests; (**B**) The line plot comparing the mean of sexual knowledge score with 95% confidence interval; (**C**) the ridge plot comparing sexual attitudes score distribution among baseline and the following two follow-up tests; and (**D**) The line plot comparing the mean of sexual attitudes score with 95% confidence interval.

Figure 2 presents the pattern of the change in sexual knowledge and attitudes scores across the three rounds of surveys. A significant increase in the scores of sexual knowledge and attitudes at the first follow-up test was observed in the intervention group, indicating positive effects of the intervention. However, the effects decreased at the second follow-up.

Table 3 shows the results of repeated measurements analysis. Positive effects of the intervention on sexual knowledge were observed in all aspects, with more pronounced effects at the first follow-up. Although positive effects of the intervention on attitudes were observed in all aspects at the first follow-up test, sustained effects were only observed at the second follow-up on attitudes toward discussing sex (β = 0.14, 95% CI 0.06–0.22) and seeking help (β = 0.17, 95% CI 0.07–0.26). The effects of the intervention on attitudes toward gender equity (β = 0.00, 95% CI −0.12–0.11), gender-based violence (β = 0.10, 95% CI −0.04–0.23), and homosexuality (β = 0.07, 95% CI −0.02–0.15) were no longer statistically significant.

To test the conclusion that the intervention is effective on students’ overall sexual knowledge and sexual attitudes at the first and the second follow-up, we calculated the maximum probability of type I error when making multiple tests. The computed *p*-value is 0.0002, which is less than 0.05. Thus, at the significant level of 0.05, we can conclude that the intervention can promote students’ sexual knowledge and attitudes at both follow-ups. The detailed procedure is included in Supplementary Materials.

The subgroup analysis by sex demonstrated similar results to the main analysis (Supplementary Material Table S3). Stronger positive effects of the intervention could be observed on sexual knowledge and attitudes scores at the first follow-up test, compared with the second follow-up test. No significant difference was observed between male and female participants.

In the sensitivity analysis, we first performed another GLM without the intervention term. No significant differences from the main analysis were detected (Supplementary Material Table S4). Then, we included only participants who remained in all rounds of the survey in the model while adjusting for the same covariates as in the main analyses. Again, no significant differences were observed (Supplementary Material Table S5).

## 4. Discussion

To our knowledge, this study is the first cluster-randomized controlled trial to assess the effectiveness of an adolescent sexuality education program in China. The findings from this study demonstrate that this internet-based and teacher-facilitated education package is effective in promoting the sexual knowledge and attitudes of adolescents in China’s resource-constrained regions. However, the findings also indicate that the effects decline over time. In particular, the intervention’s effects on attitudes on gender equity, gender-based violence, and homosexuality are not sustainable after 12 months. There are no significant differences between male and female participants.

The six middle schools involved in this study are located in Longnan, Gansu Province, where it is still economically undeveloped. A promising approach to improving the quality and accessibility of sexuality education for adolescents there is to integrate internet-based modalities into traditional school classes. The internet-based and teacher-facilitated package has several advantages over traditional sexuality education in schools, such as ease of dissemination, minimal costs after implementation, standardization of content, and lower avoidance of interventions due to stigma. In addition, compared with the use of the internet-based program alone, the inclusion of teachers ensures the complete delivery of the course modules. The inclusion of teachers in the delivery of the package also encourages teachers to study and become familiar with the contents of sexuality education. Notably, the package provides video recordings of trained educators conducting each session as a reference, which enables teachers to learn through these videos and improve their teaching skills on sexuality education. Furthermore, if the teachers were not able to deliver the courses in the package in person due to work commitments or other reasons, these videos ensure that the students can have the same standard and high-quality sexuality education as their peers.

Among the positive effects, the sustained effects on attitudes toward “seeking help when adverse outcomes happen” suggest a potential preventive impact of the sexuality education package. It appears that the sexuality education package can further prevent adverse health outcomes caused by unsafe abortions or exacerbation of STIs in participants. Moreover, there were no statistically significant differences in the effectiveness of the intervention between male and female participants, indicating that the package was effective regardless of participants’ sex, often considered as a potential effect modifier in sexuality education interventions [29].

The effectiveness of the package in this trial provides support to its application in other low resource regions in China, where adolescents are susceptible to have adverse sexual outcomes due to the lack of parents’ guidance and the neglect of sexuality education by schools [14,30]. Although the magnitude of the effect may vary due to different population characteristics such as ethnicity, the trend of the effects is expected to be similar. Although the participants were seventh-grade students aged 12–14 years old, the package was designed for middle school students and can be applied to students aged 12–18 years old. Lastly, the high proportion of eligible students whose parents or guardians consented to their participation in the intervention group suggests good acceptance of this internet-based and teacher-facilitated sexuality education for further application.

However, our study also demonstrates that the effects of the intervention on sexual knowledge and attitudes decreased after 12 months. A similar decline in the long-term effects of sexuality education on sexual attitudes was reported in other studies [9,31]. This decline may be due to the relatively short intervention period. To achieve sustainable effects in the long term, a refresher session could be conducted periodically. In addition, the package should expand its content to foster more liberal attitudes among adolescents toward gender equity, gender-based violence, and homosexuality.

There were several limitations in this study. First, because the study was based on self-reported questionnaire surveys, there is a possibility of recall bias and mismeasurement. Additionally, currently, there are no appropriate standardized questionnaire instruments concerning sexuality in children and early adolescents in the context of Chinese society. To measure the sexual knowledge and attitudes of our participants, we adopted the gender form section from the GEAS whose validity has been verified in a previous study [25]. Second, as the proportion of the participants lost to follow-up was significantly higher in the control group, compared with the intervention group, there could have been attrition bias, decreasing the comparability of the two arms. Although our use of the analysis of repeated measurements model was reported to be robust when treating randomized lost to follow-ups [32], an additional sensitivity analysis on participants who participated in all three rounds of the survey was conducted and the results of sensitivity analyses indicate no statistical differences from the main analysis. Third, due to the low prevalence of sexual behaviors among the participants, we could not assess the package’s effects on influencing adolescents’ sexual behaviors and other related sexual risks such as STIs. Lastly, because the consent should be read and signed by the participants and their guardians, there was no blinding of the participants, which may result in performance bias. This challenge often occurs in behavior intervention trials. A possible solution to control such bias is to set an additional control arm with participants receiving normal sexuality education intervention [33]. By comparing the outcomes in the normal sexuality education group and the teacher-facilitated online intervention group, performance bias could be largely controlled. Such a trial design can help researchers in future related studies.

## 5. Conclusions

In conclusion, our study demonstrates that this internet-based and teacher-facilitated sexuality education developed can be effective in promoting sexual knowledge and attitudes among adolescents in undeveloped regions in China. Although further modification is expected to ensure the sustainability of the beneficial effects, the education package can serve as a solution to the lack of sexuality education in undeveloped regions in China.

## Figures and Tables

**Table 1:** Baseline characteristics of students by intervention group.

Variable	Intervention (*n* = 264)	Control (*n* = 237)	*p*-Value
**Sex (%)**			0.668
Male	132 (50.00%)	113 (52.32%)	
Female	132 (50.00%)	124 (47.68%)	
**Age, mean (SD)**	12.9 (0.79)	12.7 (0.87)	0.012
***Hukou* (%)**			<0.001
Rural	179 (67.80%)	153 (64.56%)	
Urban	42 (15.91%)	66 (27.85%)	
Unknown	43 (16.29%)	18 (7.59%)	
**Ethnicity (%)**			0.431
Han	257 (97.35%)	234 (98.73%)	
Others	7 (2.65%)	3 (1.27%)	
**Ever had sexual practice (%)**			1.000
No	260 (98.31%)	233 (98.48%)	
Yes	4 (1.69%)	4 (1.52%)	
**Parents are divorced (%)**			1.000
No	235 (89.02%)	211 (89.03%)	
Yes	29 (10.98%)	26 (10.97%)	

**Table 2:** Baseline characteristics comparison between students remained and students lost to follow-up ^a^.

Variable	Lost to Attrition (*n* = 73)	Remained (*n* = 428)	*p*-Value
Knowledge score, mean (SD)	4.12 (2.43)	4.13 (2.57)	0.987
Attitude score, mean (SD)	7.52 (0.99)	7.91 (1.15)	0.003
Control	50 (68.49%)	187 (43.69%)	
Intervention	23 (31.51%)	241 (56.30%)	
Sex (%)			<0.001
Male	51 (69.86%)	194 (45.33%)	
Female	22 (30.14%)	234 (54.67%)	
Age, mean (SD)	13.1 (1.02)	12.8 (0.69)	0.008
*Hukou* (%)			0.388
Rural	48 (65.75%)	284 (66.36%)	
Urban	19 (26.03%)	89 (20.79%)	
Unknown	6 (8.22%)	55 (12.85%)	
Ethnicity (%) ^b^			0.645
Han	71 (97.26%)	420 (98.13%)	
Others	2 (2.74%)	8 (1.87%)	
Ever had sexual practice (%) ^b^			0.329
No	71 (97.26%)	422 (98.60%)	
Yes	2 (2.74%)	6 (1.40%)	
Parents are divorced (%)			0.002
No	57 (78.08%)	389 (90.89%)	
Yes	16 (21.92%)	39 (9.11%)	

Notes: ^a^ Difference between groups was tested by chi-square test for categorical variables and *t*-test for continuous variables, at *p* < 05; ^b^ Fisher’s exact test was used instead of chi-square test.

**Table 3:** Estimated curriculum’s effect on sexual knowledge and attitudes ^a^ (*n =* 501).

Outcome	First Follow-Up	Second Follow-Up
	Coefficient (95% CI)	Coefficient (95% CI)
**Knowledge score**	4.65 (4.12–5.17)	2.39 (1.85–2.93)
Puberty	0.99 (0.82–1.17)	0.42 (0.23–0.60)
Gender-based violence	0.64 (0.49–0.79)	0.29 (0.14–0.44)
Pregnancy and Pregnancy prevention	1.04 (0.82–1.25)	0.60 (0.38–0.82)
Abortion	0.85 (0.70–1.01)	0.60 (0.43–0.76)
Sexually transmitted infections	1.11 (0.95–1.29)	0.48 (0.31–0.65)
**Attitude score**	1.25 (1.00–1.50)	0.49 (0.23–0.75)
Discussing sex	0.26 (0.18–0.33)	0.14 (0.06–0.22)
Gender equity	0.18 (0.07–0.29)	0.00 (−0.11–0.12)
Gender-based violence	0.29 (0.16–0.42)	0.10 (−0.04–0.23)
Homosexuality	0.25 (0.16–0.33)	0.07 (−0.02–0.16)
Seeking help	0.27 (0.19–0.35)	0.17 (0.09–0.26)

Notes: ^a^ Repeated measurements model with intervention as a fixed effect term was used in the main analysis. Participants’ age, sex, hukou, ethnicity, whether ever had sexual practice, and whether parents were divorced were adjusted as fixed effect terms. Student ID and school were treated as random effect terms.

## Data Availability

Data sharing is not applicable to this article.

## Supplementary Materials

The following are available online at https://www.mdpi.com/article/10.3390/children8100885/s1, Table S1: Items used to assess participants’ sexual knowledge, Table S2: Items used to assess participants’ sexual attitudes, Table S3: Estimated curriculum’s effect on sexual knowledge and attitudes by sex, Table S4: Estimated curriculum’s effect on sexual knowledge and attitudes using GLM without intervention term, Table S5: Estimated curriculum’s effects on sexual knowledge and attitudes among students participating in all three rounds of survey (*n* = 328), Figure S1: Procedures of calculating the maximum probability of making error I type.
