# Supplementary material for: Effects of an Internet-Based and Teacher-Facilitated Sexuality Education Package: A Cluster-Randomized Trial
Source: Children (Basel). 2021 Oct 3;8(10):885. doi: 10.3390/children8100885 (PMC8534505; doi:10.3390/children8100885)
Supplement: Supplementary file 1 [file children-08-00885-s001.zip › children-1388998-supplementary.pdf]

Supplementary Materials

Table S1. Items used to assess participants' sexuality knowledge.

No	Category	Item wording	Correct Answer
1	Puberty	Male spermatorrhea (ejaculation in sleep) is a normal process of growth and development, not a disease	True
2	Puberty	Girls cannot do any sports during menstruation (Menstruation is menstrual blood discharged from the vagina)	False
3	Puberty	Masturbation (refers to stimulation of the sexual organs to achieve excitement and pleasure) is because of abnormal thoughts in the brain	False
4	Gender-based violence	Only women will encounter sexual violence	False
5	Gender-based violence	Sexual violence includes not only physical attacks, but also verbal insults, such as mocking classmates' looks	True
6	Pregnancy and contraception	Emergency contraceptives (also known as morning-after pills) have low side effects and can be used as routine contraceptive methods	False
7	Pregnancy and contraception	Safe period is a reliable method of contraception	False
8	Pregnancy and contraception	All pregnant women will have nausea, vomiting, fatigue and other physiological phenomena in the early stage	False
9	Abortion	Artificial abortion will harm women's health and may cause habitual abortion, infertility, or various types of gynecological inflammation, etc.	True
10	STD	Gonorrhea, syphilis, condyloma acuminatum and other sexually transmitted diseases must be due to unclean penetrative sex	False
11	Abortion	If one experience an unexpected pregnancy, she can just buy abortion drugs at the pharmacy	False
12	Pregnancy and contraception	Early pregnancy test paper or pregnancy test stick can accurately detect whether you are pregnant or not	False
13	STD	Among various contraceptive methods, only condoms can prevent unwanted pregnancy and the transmission of sexually transmitted diseases and HIV	True
14	STD	Only sexual transmission, blood transmission and mother-to-child transmission are the ways of HIV transmission, daily contact (eating together, shaking hands, chatting, etc.) will not be infected with HIV	True

Table S2. Items used to assess participants' sexuality attitudes.

No	Category	Item wording	More liberal Answer
1	Discussing sex	You think it is not good to talk about sex at this stage	Disagree or Strongly disagree
2	Gender	You think it's good for girls to actively pursue boys	Agree or Strongly agree
3	Gender-based violence	You feel that the person who has been sexually assaulted is not pure anymore	Disagree or Strongly disagree
4	Gender-based violence	You think that a woman being forced to have sex with her boyfriend or spouse is not considered rape	Disagree or Strongly disagree
5	Discussing sex	If there is a sexuality education course, you can study and discuss this topic in class without feeling uncomfortable	Agree or Strongly agree
6	Gender-based violence	You feel that it is acceptable to use abusive language and to beat the partner or spouse when one is angry	Disagree or Strongly disagree
7	Gender	You think boys should not show their vulnerable side	Disagree or Strongly disagree
8	Homosexuality	You think that homosexuals are abnormal and should be corrected	Disagree or Strongly disagree
9	Gender	You think it is necessary for men to be the breadwinner, and for women to be the homemaker	Disagree or Strongly disagree
10	Gender-based violence	You think girls are being sexually harassed because they dress too little or look too beautiful (Sexual harassment is sexually suggestive words, actions, touches, etc. that make you feel uncomfortable)	Disagree or Strongly disagree
11	Seeking help	If you suspect that you have a sexually transmitted disease, you will go to a regular hospital for examination	Agree or Strongly agree
12	Seeking help	If you or your sexual partner become pregnant unexpectedly, you will ask your parents for help	Agree or Strongly agree
13	Gender	Some people think that when a woman becomes pregnant, her work unit can fire her or reduce her salary. Do you agree?	Disagree or Strongly disagree
14	Homosexuality	You are willing to be friends with homosexuals	Agree or Strongly agree

Table S3. Estimated curriculum's effect on sexual knowledge and attitudes by sex[a] (N = 501).

Outcome	Male<br>Coefficient (95% CI)	Female<br>Coefficient (95% CI)	p-value
**Knowledge score**			
First follow-up	5.15 (4.38 – 5.94)	4.23 (3.52 – 4.94)	0.088
Second follow-up	2.95 (2.12 – 3.78)	2.00 (1.28 – 2.72)	0.093
**Attitude score**			
First follow-up	1.26 (0.89 – 1.63)	1.22 (0.89 – 1.55)	0.857
Second follow-up	0.65 (0.25 – 1.04)	0.43 (0.09 – 0.76)	0.397

a. Repeated measurements model with intervention as a fixed effect term was used. Participants' age, sex, hukou, ethnicity, whether ever had sexual practice, and whether parents were divorced were adjusted as fixed effect terms. Student's ID and school were treated as random effect terms.

Table S4. Estimated curriculum's effect on sexuality knowledge and attitudes using GLM without intervention term[a] (*N* = 501).

Outcome	First follow-up Coefficient (95% CI)	Second follow-up Coefficient (95% CI)
**Knowledge score**	4.61 (4.09 – 5.12)	2.36 (1.82 – 2.88)
Puberty	0.99 (0.82 – 1.17)	0.42 (0.24 – 0.60)
Gender-based violence	0.62 (0.48 – 0.75)	0.27 (0.12 – 0.41)
Pregnancy and Pregnancy prevention	1.02 (0.81 – 1.22)	0.58 (0.37 – 0.80)
Abortion	0.83 (0.67 – 0.98)	0.57 (0.41 – 0.73)
Sexually transmitted infections	1.10 (0.94 – 1.26)	0.46 (0.30 – 0.63)
**Attitude score**	1.23 (0.98 – 1.47)	0.47 (0.22 – 0.72)
Discussing sex	0.26 (0.19 – 0.34)	0.14 (0.07 – 0.22)
Gender equity	0.19 (0.08 – 0.29)	0.01 (-0.10 – 0.11)
Gender-based violence	0.27 (0.14 – 0.39)	0.07 (-0.05 – 0.20)
Homosexuality	0.25 (0.17 – 0.33)	0.08 (0.00 – 0.17)
Seeking help	0.25 (0.17 – 0.33)	0.16 (0.07 – 0.24)

a. Repeated measurements model without intervention as a fixed effect term was used in this sensitivity analysis. Participants' age, sex, hukou, ethnicity, whether had sexual practice, and whether parents were divorced were adjusted as fixed effect terms. Student's ID and school were treated as random effect terms.

Table S5. Estimated curriculum's effects on sexuality knowledge and attitudes among students participating in all three rounds of survey (*N* = 328)[a].

Outcome	First follow-up Coefficient (95% CI)	Second follow-up Coefficient (95% CI)
**Knowledge**		
Puberty	0.97 (0.78 – 1.16)	0.38 (0.19 – 0.57)
Gender-based violence	0.63 (0.47 – 0.79)	0.30 (0.14 – 0.45)
Pregnancy and Pregnancy prevention	1.05 (0.82 – 1.28)	0.61 (0.38 – 0.85)
Abortion	0.83 (0.66 – 1.00)	0.60 (0.43 – 0.77)
Sexually transmitted infections	1.09 (0.91 – 1.26)	0.46 (0.28 – 0.63)
**Attitude**		
Talking about sex	0.26 (0.17 – 0.34)	0.14 (0.06 – 0.23)
Gender equity	0.17 (0.06 – 0.29)	0.00 (-0.12 – 0.12)
Gender-based violence	0.29 (0.15 – 0.43)	0.09 (-0.04 – 0.23)
Homosexuality	0.26 (0.16 – 0.35)	0.08 (-0.01 – 0.18)
Seeking help and support	0.27 (0.19 – 0.36)	0.17 (0.09 – 0.26)

a. Repeated measurements model with intervention as a fixed effect term was used. Participants' age, sex, hukou, ethnicity, whether ever had sexual practice, and whether parents were divorced were adjusted as fixed effect terms. Student's ID and school were treated as random effect terms.

In this trial, we assessed whether the intervention is effective on students' sexual knowledge and attitudes. We have to reject both of the two null hypotheses to make the final conclusion at each follow-up.

Sexual knowledge

H_0: The intervention has no effects on students' knowledge, i.e., $\beta = 0$;

H_1: $\beta \neq 0$;

Sexual attitudes

H_0: The intervention has no effects on students' attitudes, i.e., $\beta = 0$;

H_1: $\beta \neq 0$;

Similar to the Bonferroni correction, we can calculate the maximum probability of making at least 1 type I error based on the Boole's inequality.

$$Pr\left(\bigcup_i A_i\right) \leq \sum_i Pr(A_i)$$

Estimated curriculum's effect on sexuality knowledge and attitudes ($N = 501$)

Outcome	First follow-up Coefficient	First follow-up p-value	Second follow-up Coefficient p-value	Second follow-up p-value
Knowledge score	4.65	p_1: < 0.001	2.39	p_3: < 0.001
Attitude score	1.25	p_2: < 0.001	0.49	p_4: 0.0002

The point estimate in the above table is the same as in the Table 3 in the manuscript. Approximate p-values were calculated using R package lmerTest.

Thus, the maximum probability of concluding that:

1. The intervention is effective in promoting students' sexual knowledge and sexual attitudes at the first-follow up is $p_1 + p_2 < 0.001$.

2. The intervention is effective in promoting students' sexual knowledge and sexual attitudes at the second follow-up is $p_3 + p_4 = 0.0002$.

3. The intervention is effective in promoting students' sexual knowledge and sexual attitudes at the first and the second follow-up is $p_1 + p_2 + p_3 + p_4 = 0.0002$.

Figure S1. Procedures of calculating the maximum probability of making type I error.
